# Calcifications in the infraspinatus muscle: An unusual complication of calcific tendinopathy

**DOI:** 10.1016/j.radcr.2024.11.081

**Published:** 2025-03-21

**Authors:** Zaid Ennasery, Ismail Chaouche, Salma Abouchiba, Amal Akammar, Nizar El Bouardi, Meriem Haloua, Moulay Youssef Alaoui Lamrani, Najia Hajjioui, Meryem Boubbou, Mustapha Maaroufi, Badreeddine Alami

**Affiliations:** a Department of Adult radiology, CHU Hassan II Fez, Sidi Mohammed Ben Abdellah University, Fez, Morocco; b Department of Mother and Child radiology, CHU Hassan II Fez, Sidi Mohammed Ben Abdellah University, Fez, Morocco; c Department of Physical Medicine and Functional Rehabilitation: CHU Hassan II Fez, Sidi Mohammed Ben Abdellah University, Fez, Morocco

**Keywords:** Calcific tendinopathy, Intra muscular migration, Imaging, Shoulder, Rotator cuff

## Abstract

Calcific tendinopathy is a common disorder associated with the deposition of calcium hydroxyapatite crystals within tendons. The most prevalent location is the shoulder, where it affects the tendons of the rotator cuff. The calcific phase of this condition can be divided into formative, resting, and resorptive stages. During the resorptive stage, phagocytosis of the calcific deposits may occasionally result in their extrusion. These calcifications may migrate within the tendons or into adjacent tissues, leading to local inflammation and intense pain. Intramuscular migration of calcifications, as seen in this case, is particularly rare and poses significant diagnostic challenges. It is associated with increased pain and functional limitation compared to typical cases of calcific tendinopathy. Recognizing the imaging characteristics of these uncommon complications can help prevent the need for additional unnecessary investigations and facilitate prompt intervention. In this article, we present a case involving intramuscular migration of calcifications into the infraspinatus muscle, a rare complication of calcific tendinopathy. This case involves a 55-year-old patient who was dealing with persistent and debilitating shoulder pain.

## Introduction

Calcific tendinopathy, also known as hydroxyapatite crystal deposition disease, is a condition marked by the accumulation of calcium hydroxyapatite crystals within tendons. It predominantly affects the rotator cuff, with the supraspinatus being the most frequently affected tendon (80%), followed by the infraspinatus (15%) and subscapularis (5%). Nevertheless, it can also impact sites beyond the shoulder.

Rarely, these calcifications may relocate within the shoulder. Typically, calcifications migrate to the sub-bursal space or inside the subacromion-subdeltoid bursa. Less commonly, calcifications may move to bones or muscles. Intramuscular migration, in particular, is an unusual and underreported complication that can significantly alter the clinical presentation and management of calcific tendinopathy.

The exact etiology of calcific tendinopathy remains elusive; however, several factors have been implicated. These include low oxygen levels within the tendon, hormonal influences (particularly in premenopausal women), and mechanical stresses.

The pathophysiology of calcific tendinopathy involves distinct stages, including precalcific, calcific, and postcalcific phases. During the resorptive phase, calcifications may migrate to neighboring tissues, as observed in our case. This phase, which is often associated with severe pain, can result in complications such as intramuscular migration, which heightens both diagnostic and therapeutic challenges.

Clinically, calcifying tendinopathy may exhibit no symptoms, but it can sometimes lead to pain and mechanical issues, with the resorptive phase often being the most painful. Diagnosis typically involves radiography to reveal the extent, shape, and contour of the calcification. Ultrasound is also a highly effective imaging method for assessing calcifying tendinopathy, offering a detailed view of calcifications within appendicular tendons and accurate monitoring of different stages.

Other imaging tools like CT and MRI are generally unnecessary unless osseous or muscular involvement is suspected. MRI is especially valuable for evaluating bone and muscular edema. In cases of intramuscular migration, MRI is pivotal in distinguishing between tendon-based and muscle-based complications, providing detailed anatomical insights.

Various treatments for Calcific tendinopathy are available, favoring conservative approaches initially, including rest, nonsteroidal anti-inflammatory drugs, physical therapy, and corticosteroid subacromial infiltrations in later stages. Surgery is considered only if conservative treatments prove ineffective. However, in rare cases of intramuscular migration, the severity of pain and the functional limitation may prompt earlier consideration of surgical intervention.

## Case presentation

Our patient is a 55-year-old woman, that consulted with complaints of persistent shoulder pain in her right shoulder. She reported that the pain had been worsening over the past few months limiting her ability to perform daily activities. Ms D.Z denied any history of trauma to the shoulder, she noted that the pain seemed to be exacerbated with certain movements such as reaching overhead or lifting objects.

The patient described the pain as an ache that radiated from the top of her shoulder down to her upper arm. She also reported occasional episodes of sharp stabbing pain during certain movements. this pain sometimes interfered with her ability to perform tasks that required overhead reaching or lifting.

Physical examination showed tenderness over the supraspinatus and infraspinatus tendon insertions. Active and passive range of motion of the right shoulder was limited, especially in abduction and external rotation. The patient exhibited signs of muscle weakness and atrophy of the right shoulder.

The radiograph of the right shoulder reveals evidence of calcific tendinopathy in the infraspinatus tendon; it showed irregular calcifications within the soft tissues adjacent to the greater tuberosity of the humerus corresponding to the expected location of the infraspinatus tendon insertion. they appear as radiopaque densities with well-defined margins. No evidence of bony erosion or cortical irregularities is appreciated (Fig. A).

**A:**
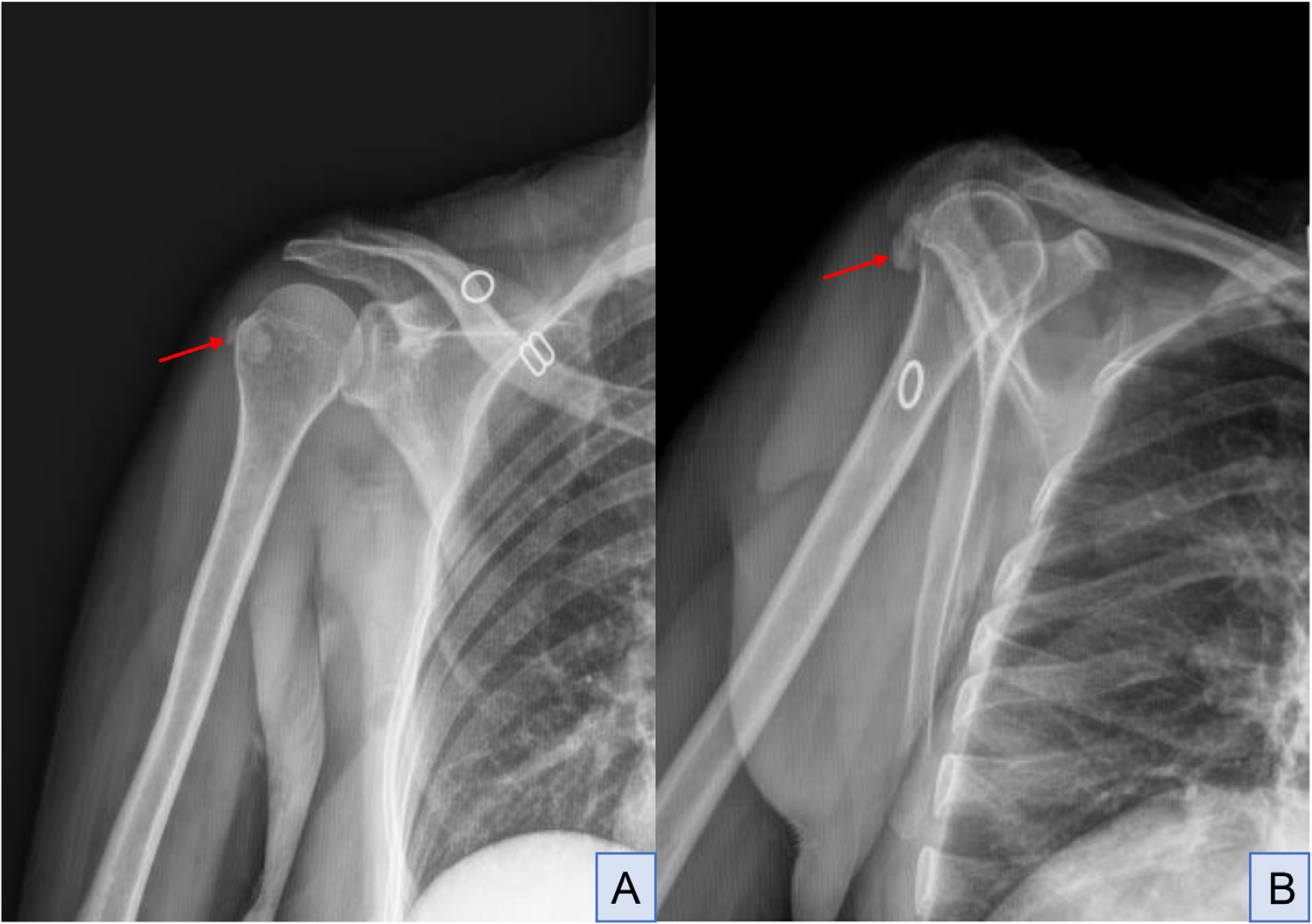
Full-face (A) and internal-rotation (B) radiograph; radiopaque calcifications (red arrow) within the soft tissues adjacent to the greater tuberosity of the humerus.

The presence of calcifications in the soft tissues surrounding the greater tuberosity suggests an active phase of the disease process, needing further evaluation with ultrasound and MRI to assess for associated inflammatory changes and intra-muscular migration of calcifications.

Ultrasound (US): The ultrasound examination revealed evidence of calcific tendinopathy in the infraspinatus tendon characterized by hyperechoic foci with acoustic shadowing (Fig. B).

**B:**
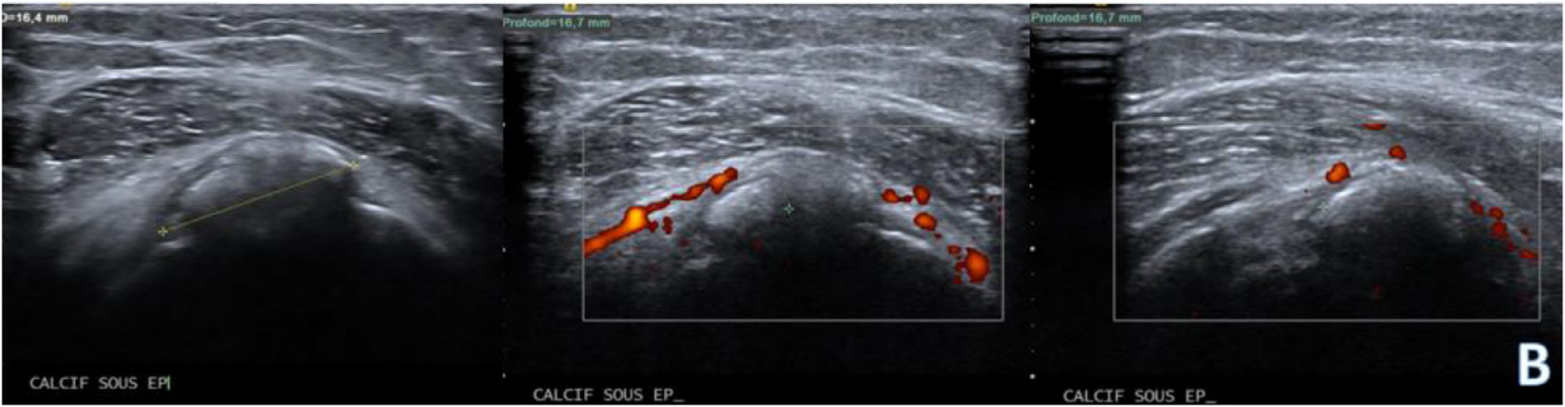
Shoulder ultrasound mode B; hyperechoic foci with acoustic shadowing in the infraspinatus tendon with increased vascularity within the infraspinatus muscle belly extending distally towards the teres minor muscle.

Dynamic scanning during shoulder movement demonstrated incomplete gliding of the infraspinatus tendon caused by the calcifications, there was also evidence of an inflammatory flare-up with increased vascularity and localized edema within the infraspinatus muscle belly extending distally towards the teres minor muscle.

MRI of the right shoulder confirmed the presence of calcific tendinopathy in the infraspinatus tendon, with multiple calcifications measuring up to 15 mm for the largest one. This calcification had migrated distally into the belly of the infraspinatus muscle resulting in significant edema and inflammation. The edema was characterized by moderate T2 hyperintensity extending towards the teres minor muscle. This was indicative of an acute exacerbation of calcific tendinopathy, MRI also revealed evidence of nonfissured tendinopathy in the subscapularis tendon, characterized by tendon thickening and signal abnormalities consistent with chronic degenerative changes. Additionally, a minimal joint effusion was observed in the glenohumeral joint, suggestive of mild synovitis or joint irritation (Fig. C-D-E).

**C-D-E:**
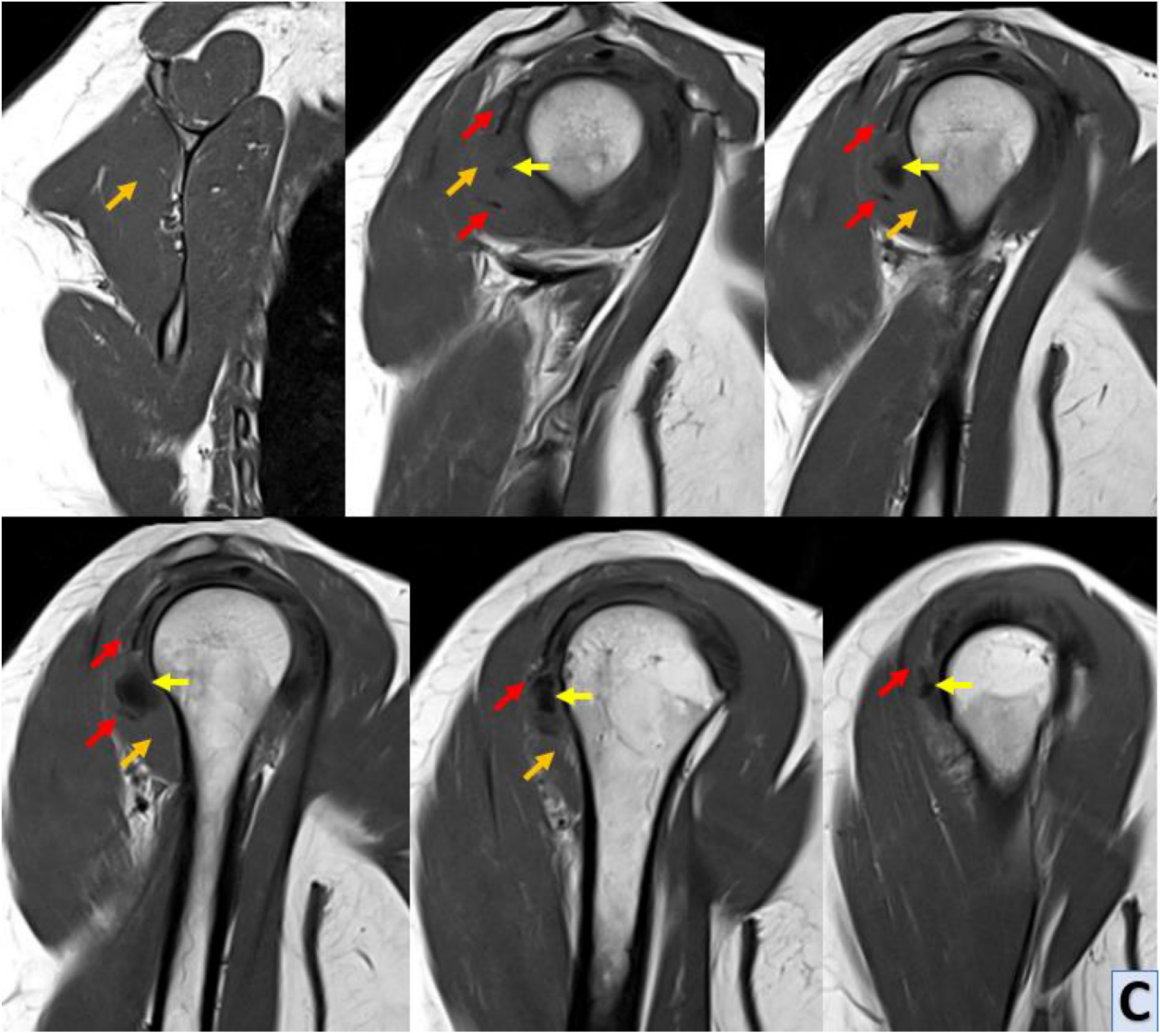

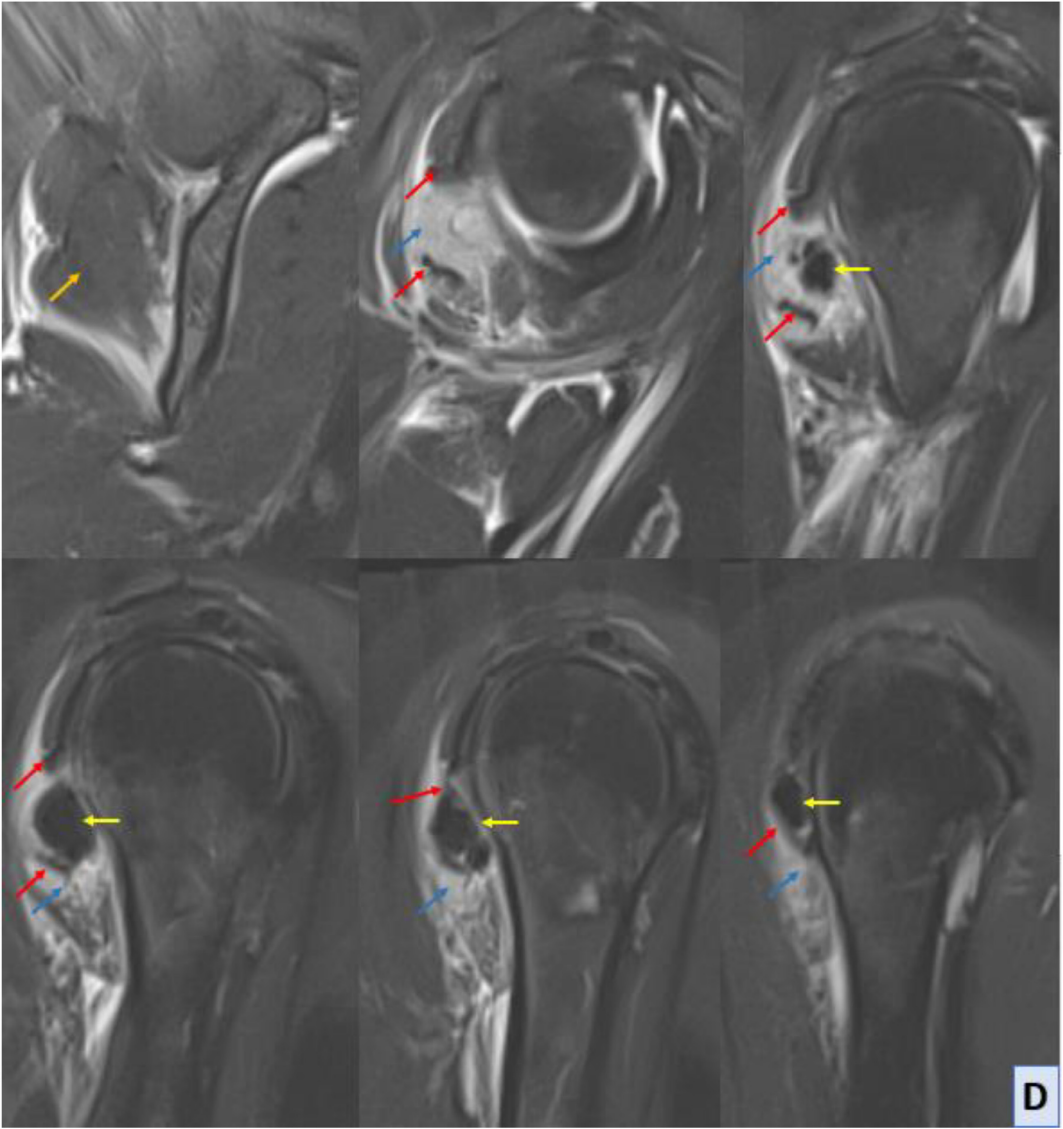

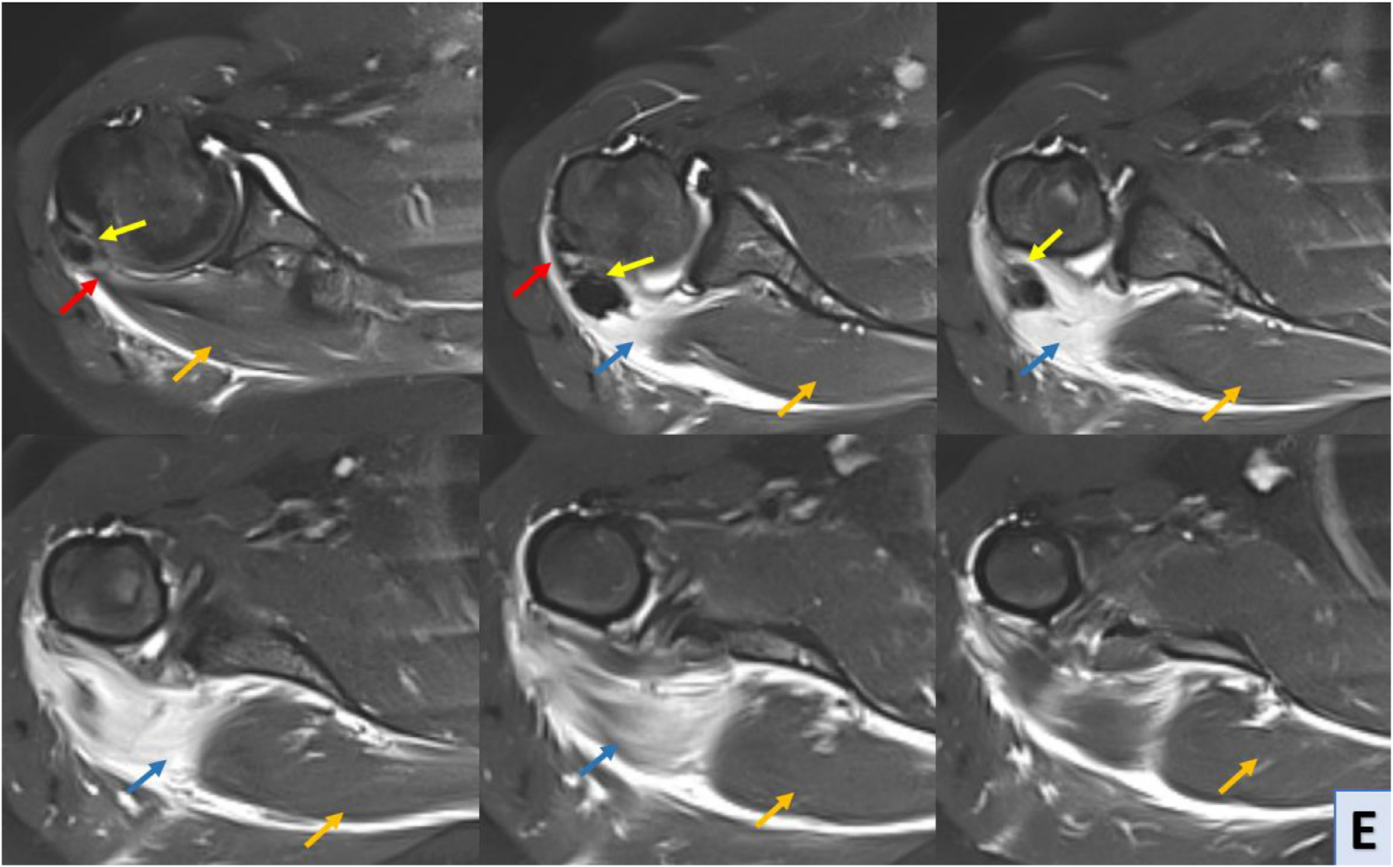
Shoulder MRI with sagittal T1 sequence (C), sagittal Blade DP FS (D) and axial Blade DP FS (E) sequences; MRI reveals asignal calcified images (yellow arrow) within the distal infraspinatus tendon (red arrow), and deeply and distally in the belly of the infraspinatus muscle. This intramuscular calcification is associated with hypersignal muscle edema (blue arrow), contrasting with the normal infraspinatus muscle (orange arrow).

Based on the clinical presentation and imaging findings, Ms. B.M was diagnosed with intramuscular migration of calcific tendinopathy in the infraspinatus of the right shoulder.

Ms. B.M was managed conservatively with a combination of rest, nonsteroidal anti-inflammatory drugs (NSAIDs), and physical therapy. Follow-up showed progressive improvement in her symptoms during evaluations.

## Discussion

Calcific tendinopathy, or hydroxyapatite crystal deposition disease, manifests as the accumulation of calcium hydroxyapatite crystals within tendons, which primarily affects the rotator cuff [1,2].

The prevalence of this condition is quite common, with the supraspinatus tendon being the most frequently involved (approximately 80% of cases), the infraspinatus tendon is affected in about 15% of cases, while the subscapularis tendon is affected in only 5% of cases [3].

Despite the typical localization within the rotator cuff, calcific deposits may migrate within different anatomical spaces of the shoulder complex leading to various clinical presentations and diagnostic challenges. Common migration patterns involve relocation to the sub-bursal space or the subacromion-subdeltoid bursa, where they can cause local inflammation and pain. Calcific deposits can rarely migrate to adjacent bones or muscles further complicating the clinical picture [[1], [2], [3]].

The cause of rotator cuff calcific tendinopathy is not completely understood but is likely associated with low oxygen levels within the tendon, hormonal factors also potentially contributing given the higher incidence in premenopausal women [4].

Uhthoff and Loehr's classification of calcific tendinopathy provides a comprehensive framework for understanding the pathophysiological stages of this condition. They defined 3 distinct stages of this pathology: precalcific, calcific, and postcalcific. The calcific phase can be further categorized into formative, resting, and resorptive stages [5].

The precalcific phase marks the initiation of calcification within an intact tendon; This phase is characterized by cellular changes that precede the visible deposition of calcium hydroxyapatite crystals. Fibrocartilaginous metaplasia, likely induced by low oxygen levels within the tendon, triggers the formation of calcic deposits. Following the precalcific phase, the calcific stage unfolds, encompassing formative, resting, and resorptive stages. During the formative stage, homogeneous and well-defined calcifications emerge within the tendon, representing the maturation of the initial fibrocartilaginous metaplastic changes. As the process progresses into the resting stage, the calcifications remain stable and exhibit minimal changes in size or appearance. The resorptive stage marks a dynamic phase characterized by cellular activity surrounding the calcific deposits. Increased vascularity within the tendon indicates an important inflammatory response aimed at resorbing the calcifications., this phase is also associated with the potential for calcific deposits to migrate to neighboring tissues. While the exact mechanism underlying migration remains unclear, mechanical forces play likely a role during the resorptive phase, contributing to the displacement of calcium hydroxyapatite crystals. In the postcalcific stage, the tendon undergoes self-healing and repair processes aimed at restoring tissue integrity. Granulation tissue enriched with fibroblasts initiates the remodeling of the tendon at the site of previous calcific deposits. Over time, fibroblasts and collagen align along the long axis of the tendon, facilitating the restoration of its structural integrity [[4], [5], [6], [7], [8]].

Symptoms in calcifying tendinopathy can range from asymptomatic to debilitating. The resorptive phase, characterized by increased vascularity and inflammation around calcific deposits often induces intense pain, which can be sharp and localized. Patients may experience limitations in shoulder mobility and function, especially during activities that involve overhead movements or rotation of the arm. Pain may also disrupt sleep and lead to functional impairment in daily tasks. Additionally, mechanical issues such as crepitus or clicking sensations might accompany the pain, further affecting the quality of life [[5], [6], [7], [8]].

Calcific tendinopathy poses a diagnostic challenge due to its varied presentations and potential complications, including intra-muscular migration of calcific deposits. Imaging modalities such as conventional radiography, ultrasound, and MRI play crucial roles in accurately diagnosing and characterizing this condition, each offering unique advantages in the evaluation of calcific tendinopathy and its associated complications.

Conventional radiography serves as the initial imaging modality for evaluating calcific tendinopathy, providing informations about the size, shape, and location of calcifications within tendons. In the context of intra-muscular migration, radiography can demonstrate the presence of calcifications within the muscle belly or adjacent bones that typically appear as well-defined dense opacities on radiographs, conventional radiography may however have limitations in detecting subtle soft tissue changes or intramuscular migration, necessitating additional imaging modalities for comprehensive evaluation [9].

Ultrasound is a dynamic and noninvasive imaging modality widely used for assessing calcific tendinopathy and its complications. It offers real-time visualization of calcifications within tendons and muscles, allowing for detailed characterization of their morphology and distribution. In cases of intra-muscular migration, ultrasound can accurately depict the extent of calcifications within the muscle belly and surrounding soft tissues. Color Doppler ultrasound can demonstrate associated hyperemia, indicative of inflammation and tissue damage. Ultrasound is particularly valuable for guiding interventions such as needle aspiration or corticosteroid injections, offering precise targeting and monitoring of treatment response [10,11].

Magnetic resonance imaging (MRI) provides comprehensive anatomical and pathological information, offering superior soft tissue contrast and multiplanar imaging capabilities. MRI can delineate the extent of soft tissue changes, including edema, inflammation, and intramuscular migration of calcific deposits. T1-weighted sequences typically show intensity low signal calcifications within tendons and muscles, while T2-weighted sequences may reveal surrounding edema and inflammation as areas of increased signal intensity. MRI can also identify associated complications such as tendon tears or joint effusions, providing valuable information for treatment planning [12].

Treatment of calcific tendinopathy varies depending on the stage of the disease and the presence of complications such as intramuscular migration.

Conservative measures, including rest, NSAIDs, physical therapy, and ultrasound-guided corticosteroid injections, are often effective in managing mild cases [13,14].

In refractory cases or those with significant complications such as tendon tears or intramuscular migration, surgical intervention may be necessary. Arthroscopic debridement and removal of calcific deposits are commonly performed, aiming to alleviate symptoms and restore function [15].

Multidisciplinary collaboration involving orthopedic surgeons, radiologists, and physical therapists is essential for optimizing treatment outcomes and minimizing the risk of disease recurrence.

## Conclusion

Calcific tendinopathy of the shoulder complicated by muscular migration of calcific deposits presents a challenging clinical scenario. Detailed imaging evaluation, including ultrasound and MRI, played a crucial role in confirming the diagnosis and guiding treatment decisions. While conservative measures may provide temporary relief, surgical intervention is often necessary for definitive management. Multidisciplinary collaboration among orthopedic surgeons, radiologists, and physical therapists is essential for optimizing patient outcomes and facilitating return to functional activities.

## Patient consent

I, the author of the article *«*Calcifications in the infraspinatus muscle, an unusual complication of calcific tendinopathy*»,* declare that informed written consent was obtained from the patient for publication of the case report and all imaging studies in RADIOLOGY CASE REPORTS.
